# Tremor of the Eyes, or of the Head, in Parkinson's Disease?

**DOI:** 10.1002/mds.25478

**Published:** 2013-04-29

**Authors:** R John Leigh, Susana Martinez-Conde

**Affiliations:** 1 Neurology Service, Veterans Affairs Medical CenterCleveland, Ohio, USA; 2 Department of Neurology, Case Medical Center, Case Western Reserve UniversityCleveland, Ohio, USA; 3 Department of Neurobiology, Barrow Neurological InstitutePhoenix, Arizona, USA

Recently, Gitchel et al.^1^ reported that “pervasive ocular tremor” is present in patients with Parkinson's disease (PD) while they attempt steady visual fixation. This was a universal finding in the 112 PD patients that they studied, but was generally absent from their 60 healthy control subjects. This finding of a pervasive ocular tremor in PD has generated substantial interest but also controversy, as is evident by the article by Kaski et al.,^2^ and the ensuing correspondence by Baron et al.^3^. Because pervasive ocular tremor might provide a most useful biomarker for PD, it seems justified to examine the validity of the measurements of Gitchel et al.^1^ and consider alternative explanations for their results, before they gain wide acceptance and application. Here we summarize some of the evidence for and against the case for pervasive ocular tremor in PD, and suggest future experiments to resolve conflicting opinions.

First, consider the visual requirements of eye movements during attempted fixation of a stationary, high-contrast visual target, such as a letter X. One requirement is that the image of the target of interest must be held on the foveal region of the retina (an area subtending about 0.5 degrees in diameter, which has the highest visual acuity).^4^ An additional requirement for clear vision is that the image must not move more than about 5 degrees/s over the retina, or visual acuity will decline and observers will perceive illusory motion of the target (oscillopsia).^5^

Second, consider how well normal healthy subjects can hold their gaze still when they visually fixate a small, stationary target and their head is immobilized (best achieved using a custom-made bite-bar). During such conditions, a subject's gaze (line of sight with respect to the earth) is not perfectly still, but is disrupted by 3 distinct perturbations: microtremor, microsaccades, and drifts of the eyes.^6^,^7^ Microtremor has dominant frequencies averaging approximately 84 Hz and ranging from 70 Hz to 103 Hz. Due to its high frequency (much greater than frequencies achieving perceptual flicker fusion) and very small amplitude (∼1 photoreceptor width, <0.5 arcmin),^8^ microtremor is thought to have little influence on vision. Microsaccades are rapid movements, typically less than 1 degree in size with frequencies of 1 to 2 Hz, which appear to play an important role in counteracting perceptual fading during fixation.^9^,^10^ Recent work suggests that square-wave intrusions, so common in patients with movement disorders, lie on a continuum with microsaccades.^11^,^12^ Smooth intersaccadic drifts during attempted fixation are thought to be under the control of smooth eye movements,^13^ and typically do not exceed 0.1 degrees/s, unless visual feedback is interrupted, for example, by switching the environment to darkness.^14^ When all these “fixational eye movements” are taken into account, the standard deviation of gaze is typically <0.2 degrees, so the eyes are not perfectly still, but the image of an object of interest spends most of its time on the fovea. (One must keep in mind the possibility of foveal visual impairment in PD, however, which might affect fixation behavior.^15^) If the subject's head is now free to move, gaze stability also depends on the vestibulo-ocular reflex, which compensates (although not perfectly) for head perturbations. If the vestibulo-ocular reflex is absent, even transmitted cardiac pulsations can disrupt vision, because the direction of gaze now depends on head position.^16^ Blurred vision and oscillopsia also occur when abnormal eye movements, such as acquired pendular nystagmus, induce retinal image motion that exceeds 5 degrees/s.^17^

With this background, we can now ask: What manner of eye movements have Gitchel et al.^1^ discovered to be present in every Parkinsonian patient whose gaze they measured during visual fixation? They report small (mean, 0.27 degrees), high-frequency (mean, 5.7 Hz) oscillations of the eyes, with greater vertical than horizontal components. Although these eye movements were small enough not to displace the targets image from the fovea, their high frequency meant that their root-mean-square velocity exceeded 5 degrees/s, which would be expected to induce oscillopsia. Does the reported pervasive ocular tremor of PD correspond to any known form of eye movement occurring during attempted visual fixation in healthy subjects? The reported frequencies of ocular tremor of PD would appear to exclude both microsaccades (too high) and microtremor (too low), while the strong oscillatory nature of the motion and its moderate velocity would rule out drifts. Thus, there is no straightforward correspondence between pervasive tremor and any of the fixational eye movement types known in normal vision.

Further, a singular difference between pervasive tremor of PD and other known eye movements is that PD eye tremor was reportedly unaffected by “saccades, blinks or other eye movements.”^1^ Since probably every other form of ocular oscillation has been reported to be influenced by saccades, gaze angle, convergence, or vestibular stimuli,^17^ more studies are required to clarify this point. Lack of such influence would point to a source for PD pervasive ocular tremor outside of the ocular motor system, such as head tremor (transmitted limb tremor, in some patients), which is reported to be typically about 4 to 5 Hz in PD.^18^ Kaski et al.^2^ address this issue in their article and demonstrate how head oscillations in 2 patients with PD can be held at least partly responsible for their ocular oscillations, eye velocity being 180 degrees out-of-phase with head velocity, and the magnitude of eye oscillations being dependent on the amplitude of the head oscillations. In conflict with this result, Gitchel et al.^1^ measured eye and head movements in 62 of their PD patients and in 31 control subjects and found that “head movements did not contribute to the ocular instability findings.” Could the differences between these 2 studies be due to different methodologies? Certainly measuring eye and head rotations with 2 different types of device poses difficulties of calibration and synchronization between the 2 records. Further, the limitations of optical tracking techniques (such as the system used by Gitchel et al.^1^) to characterize slow, small eye movements during attempted fixation have been noted in a recent primate study, even though the animals' heads were immobilized with chronically implanted headposts.^19^

**FIG. 1:**
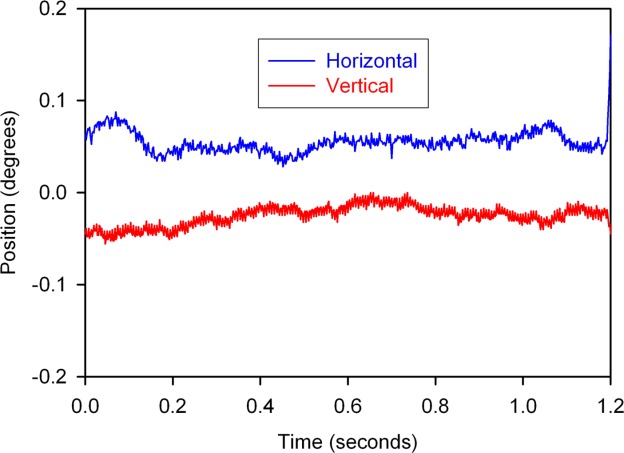
Representative record of left eye position-in-space (gaze) during binocular visual fixation of a stationary target by a 68-year-man with 13-year duration, DOPA-responsive Parkinson's disease, with Hoehn-Yahr score of 2. Horizontal and vertical records have been offset to aid clarity of display. He had no observable head tremor and no ocular tremor is apparent on the record. The record was made using the magnetic-field/search-coil technique, with analog filtering of coil signals (bandwidth, 0–150 Hz) before digitization at 500 Hz. No digital filtering was used; details of this methodology have been described.^24^,^29^

Another problem is that it is difficult to completely immobilize subjects' heads even with a custom-made bite-bar, which is often uncomfortable for the subject. One technology that does lend itself to a direct comparison of eye and head rotations is the magnetic-field/search-coil approach, for which the same device is used to record both variables with a sensitivity of <0.05 degrees, and a broad linear range and bandwidth.^20^ When the field coils are large (2 m), an additional advantage of this technique is that the eye coil measures gaze (eye position in spatial coordinates), which is indifferent to head tremor, unless the vestibulo-ocular reflex is deficient.^17^ Using this approach we have not encountered ocular tremor during our studies of parkinsonian patients over the past 30 years^21^–^25^; Figure 1 provides an example. However, it should be noted that we have not systematically studied fixation stability in as large a number of PD patients as have Gitchel et al.,^1^ and a prospective study is required to test our impression.

Indeed, the power of the study by Gitchel et al.^1^ rests on the large number of patients that they studied and the curious finding that fixation instability was present in *every* one of their 112 PD patients and only 2 of the control subjects (1 of whom subsequently developed PD). This unanimity seems surprising given that the literature contains dozens of studies of eye movements in patients with PD using reliable methods, including a substantial number aimed at fixation behavior, yet only Gitchel et al.^1^ and another small study^26^ noted ocular tremor (both under conditions in which patients' heads were not immobilized).

Another reason why the presence of ocular tremor in PD is surprising is that it is not evident clinically. Patients with PD do not complain of oscillopsia generally, but this would be expected in at least some patients if retinal image velocity exceeds 5 degrees/s. Rare PD patients who do complain of oscillopsia are reported to have a deficient vestibulo-ocular reflex and head tremor.^27^ Finally, neurologists who view their patient's optic discs with an ophthalmoscope know how sensitive this method is for detecting eye movements (as small as 0.1 degrees; eg, microflutter)^28^; the pervasive ocular tremor described by Gitchel et al.^1^ should, therefore, be visible.

Thus, more work is required by other laboratories to investigate visual fixation behavior in a large cohort of PD patients and control subjects, and systematically study the properties of any ocular tremor that is detected. For such a study, the magnetic field/search coil approach would appear to be optimal, but other techniques may be equally useful, provided calibration and synchronization issues are properly addressed, and the eye tracker is insensitive to movements of the device with respect to the subject's head. In selected PD patients thought to have ocular tremor, the effect of immobilizing their head on a custom-made bite-bar would provide a valuable comparison. Finally, the community has a good deal to be grateful to Gitchel et al.^1^ for shaking up the field; even if it turns out that pervasive ocular tremor in PD arises from head motion compensation, their report may promote a reappraisal of head tremor in this disorder. But to resolve this controversy, input from laboratories that study eye movements in patients with PD is needed: Has anyone encountered “pervasive ocular tremor”?
